# State of the Science for Kidney Disorders in Phelan-McDermid Syndrome: UPK3A, FBLN1, WNT7B, and CELSR1 as Candidate Genes

**DOI:** 10.3390/genes13061042

**Published:** 2022-06-10

**Authors:** Megan D. McCoy, Sara M. Sarasua, Jane M. DeLuca, Stephanie Davis, Katy Phelan, Roger Curtis Rogers, Luigi Boccuto

**Affiliations:** 1 Healthcare Genetics Program, School of Nursing, Clemson University, Clemson, SC 29634, USA; mmccoy2@g.clemson.edu (M.D.M.); jdeluca@clemson.edu (J.M.D.); stephad@clemson.edu (S.D.); lboccut@clemson.edu (L.B.); 2 Florida Cancer Specialists & Research Institute, Fort Myers, FL 33908, USA; kphelan@flcancer.com; 3 Greenwood Genetic Center, Greenville, SC 29605, USA; crogers@ggc.org

**Keywords:** Phelan-McDermid syndrome, 22q13.3 deletion syndrome, kidney disorders, urogenital anomalies

## Abstract

Phelan-McDermid syndrome (PMS) is a neurodevelopmental disorder caused by chromosomal rearrangements affecting the 22q13.3 region or by *SHANK3* pathogenic variants. The scientific literature suggests that up to 40% of individuals with PMS have kidney disorders, yet little research has been conducted on the renal system to assess candidate genes attributed to these disorders. Therefore, we first conducted a systematic review of the literature to identify kidney disorders in PMS and then pooled the data to create a cohort of individuals to identify candidate genes for renal disorders in PMS. We found 7 types of renal disorders reported: renal cysts, renal hypoplasia or agenesis, hydronephrosis, vesicoureteral reflux, kidney dysplasia, horseshoe kidneys, and pyelectasis. Association analysis from the pooled data from 152 individuals with PMS across 22 articles identified three genomic regions spanning chromosomal bands 22q13.31, 22q13.32, and 22q13.33, significantly associated with kidney disorders. We propose *UPK3A*, *FBLN1*, *WNT7B*, and *CELSR1*, located from 4.5 Mb to 5.5 Mb from the telomere, as candidate genes. Our findings support the hypothesis that genes included in this region may play a role in the pathogenesis of kidney disorders in PMS.

## 1. Introduction

Phelan-McDermid syndrome (PMS) is a disorder frequently displaying neonatal hypotonia, seizures, and neurological deficits, such as global developmental delay, moderate to severe intellectual impairment, absent or severely delayed speech, and autism or autistic-like behavior [1]. Kidney disorders are among the most frequent non-neurological features of the syndrome, being reported in up to 40% of cases of PMS [2,3,4,5], but few studies have characterized them in depth or investigated candidate genes in the 22q13 region. The disorder is caused by pathogenic variants in the *SHANK3* gene or deletions along chromosome 22q13 due to terminal deletions, balanced or unbalanced translocations, interstitial deletions, or ring chromosomes. *SHANK3* is almost always included in the deletion, although other genes are often involved in the deletions as well. Pathogenic variants of *SHANK3* alone have been shown to be associated with several neurobehavioral features of PMS [6]. Haploinsufficiency of *SHANK3* results in disruption of synaptic function, leading to many of the neurological features of the syndrome. *SHANK3* is unlikely to be associated with renal phenotypes as no cases of renal disorders were observed in a large study of PMS caused by *SHANK3* pathogenic variants or *SHANK3* microdeletions [6]. The 22q13 genes deleted in PMS that lead to renal disorders remain unknown.

The types of kidney disorders reported in the literature include renal cysts, renal hypoplasia or agenesis, hydronephrosis, vesicoureteral reflux, kidney dysplasia, horseshoe kidneys, and pyelectasis [2,3,5,7,8,9,10,11,12,13,14,15,16,17,18,19,20,21]. Renal cysts are pouches of fluid of varying sizes that are diagnosed through ultrasound and eventually drained or surgically removed when necessary [22,23]. The prevalence of renal cysts in the general population is 27% and increases with age [24]. Renal agenesis is the absence of one or both kidneys, due to failure to develop. In the general population, unilateral agenesis occurs at 1:800–1200 of all births and is more common than bilateral agenesis, which occurs in 1:10,000 births [25,26]. Hypoplasia is defined by smaller sized kidneys with normal, but fewer numbers of nephrons. Both hypoplasia and agenesis are diagnosed via ultrasound, and the treatment options depend on the presence of at least one functioning kidney [25,26]. Hydronephrosis, diagnosed by ultrasound, is enlargement of the kidneys due to excessive collection of urine [27]. Hydronephrosis is not believed to be hereditary and is detected in 1–5% of pregnancies [28]. Vesicoureteral reflux is diagnosed through a cystogram test showing an abnormal flow of urine from the bladder upstream into the ureters; it occurs in approximately 1:100 infants in the general population [29,30]. Although this disorder usually reverses spontaneously, it can place the patient at a higher risk for urinary tract infections. Kidney dysplasia is abnormal development and differentiation of the internal structures of one or both kidneys and can be detected on ultrasound [31]. Dialysis or kidney transplant are recommended when both kidneys are impaired, whereas no treatment is necessary when a functioning kidney is present [32,33]. Horseshoe kidney is diagnosed through ultrasound, and other imaging modalities, as the fusion of the two kidneys into a single, U-shaped organ, and requires no treatment. Horseshoe kidney occurs in approximately 1:400–600 individuals in the general population, with onset occurring during fetal development as the kidneys move into their normal position in the dorsal region [34]. Pyelectasis is a dilation of the renal pelvis which may be the result of urine being unable to flow freely from the kidney to the bladder, or from urine backing up from the bladder into the kidneys [35]. Pyelectasis is a relatively common ultrasound finding in fetuses, with males being affected three times more frequently than females. In most cases, pyelectasis resolves spontaneously, having no subsequent ill effects on the baby. However, the detection of prenatal pyelectasis should prompt a scan for other renal anomalies as well as a test for the presence of genetic abnormalities [35,36]. Horseshoe kidney, pyelectasis, kidney dysplasia, and renal agenesis/hypoplasia are congenital, whereas renal cysts, hydronephrosis, and vesicoureteral reflux can occur both at birth or later.

The high estimated prevalence and clinical severity of these disorders merits an investigation into their genetic cause. Further, understanding the genes responsible for these disorders has the potential to assist with identifying at-risk patients in need of diagnostic workup and determining treatment; it can also reveal pathogenic mechanisms that may lead to isolated kidney disorders outside of PMS. Most studies of PMS focus on neurobehavioral features rather than kidney disorders, even when data on these features were collected, leaving valuable data about the renal system unexamined. Additionally, these extant studies frequently have an inadequate sample size to conduct statistical analyses. Therefore, the authors conducted a systematic review of the literature and pooled the published reports to create a large cohort of individuals assessed for the presence of kidney disorders to identify genes or genomic regions on 22q13 that may contribute to a discernible renal phenotype in PMS.

## 2. Materials and Methods

A systematic literature review was performed [updated to February 2022] to identify individuals with PMS evaluated for kidney disorders in Google Scholar and PubMed using the following keywords: [“Phelan-McDermid” OR “22q13.3 deletion” OR “monosomy 22q13” OR “telomeric 22q13” OR “*SHANK3*” OR “ring 22” OR r(22)] AND syndrome, “22q13.3 terminal deletion” OR “22q13.3 deletion” “22q13.3” AND “terminal deletion”, “kidney disorder” OR “renal”, “dysplastic kidneys” OR “horseshoe kidneys” OR “pyelectasis” OR “renal cysts” OR “renal agenesis” OR “hypoplasia” OR “hydronephrosis” OR “vesicoureteral reflux”, “bladder disorder” OR “urinary disorder”. Information was collected on all cases where individuals were evaluated for kidney disorders, regardless of whether a renal disorder was present. Chromosomal breakpoints, genome build, deletion sizes, type of rearrangement, type of kidney disorder if present, and sex of the individual were abstracted from the literature. All manuscripts related to kidney disorders in relation to PMS were included. Manuscripts were excluded if they reported only animal model data or were not published in English. The data from individual reports were pooled for analysis. This process followed the methods of Samogy-Costa et al., who were the first to use a pooled analysis for kidney disorders in PMS and suggested an association between deletion size and kidney disorders [4]. All genetic data not originally reported as hg19 were converted to the hg19 genome build coordinates using the UCSC Genome Browser LiftOver tool [37]. In cases where terminal deletion size was provided instead of chromosomal breakpoints, the breakpoints were estimated by subtracting the reported deletion size from the terminus of the euchromatic portion of chromosome 22 set to position 51244566. Individuals missing genomic deletion information were excluded from the association analyses between genomic position and kidney disorder. Individuals were classified as not having kidney disorders if they were reported to not have a kidney disorder or their abdominal ultrasound was normal.

### 2.1. Statistical Analysis

The 2-sided student’s *t*-test was used to compare mean deletion sizes for patients with or without kidney disorders using Excel software. Categorical variables were compared using a 2-tailed uncorrected chi-square using OpenEpi.com. A *p*-value < 0.05 was considered statistically significant.

Association mapping was performed between kidney disorders and the segmental copy number variant (CNV) data across chromosome 22q13 using the PLINK toolset v. 1.9 software (http://pngu.mgh.harvard.edu/purcell/plink/) [38]. Empirical *p*-values were calculated based upon 50,000 permutations and adjusted for multiple testing to identify the genomic regions most associated with kidney disorders. Regional tests were performed to identify genes within the segmental CNV data using the gene coordinates provided in the software resources for hg19. *p*-values < 0.05 were considered statistically significant, and, for visualization purposes, −log10(*p*-value) was plotted against the genomic position to identify the regions most strongly associated with kidney disorders.

### 2.2. Candidate Gene Prioritization

Several criteria were used to identify candidate genes within the genomic regions identified as being statistically significantly associated with kidney disorders. One tool was the use of ToppGene suite [39] to prioritize genes based on a training set of 31 known kidney-related genes [22,26,31,40,41,42] (Supplemental Table S1) and the test set of genes within the CNV segments associated with kidney disorders at *p* < 0.05. Molecular function, biological process, cellular component, human phenotype, pathway, and disease were the features used to prioritize candidates. Other tools used were haploinsufficiency predictions from Huang et al. 2010 [43] and pLI scores from gnomAD [44].

## 3. Results

### 3.1. Renal Disorders Assessed in Phelan-McDermid Syndrome Cohorts

Ten cohorts of individuals with PMS were presented in the literature with at least one individual reported to have a kidney disorder (Table 1) [2,3,4,5,7,9,15,21,45,46]. The percentage of individuals with kidney disorders ranged from 9% to 40% (mean of 22% across the ten studies), with cohort sizes ranging from 9 to 148. Bonaglia et al. (2011) looked at a cohort of n = 44 to investigate the poorly understood molecular and cellular mechanisms of terminal deletions in PMS [7]. Within this cohort, 10% of patients were found to have kidney disorders. These include two cases of hydronephrosis and one case each of renal agenesis and renal hypoplasia. Jefferies et al. (2005) found 17% of individuals with renal abnormalities in their cohort of 30 cases, presenting with urinary tract infections (n = 1), vesicoureteral reflux (n = 3), and multicystic kidney (n = 1) [15]. Sarasua et al. (2014) found vesicoureteral reflux in 14% (18/133) of individuals, polycystic kidneys in 5% (6/132), pyelectasis in 5% (7/129), and hydronephrosis in 9% (11/126) [3]. Tabet et al. (2017) examined a cohort of 78 individuals, with seven of them (9%) having renal disorders, including one case each of multicystic kidneys, renal dysplasia, kidney duplication, hydronephrosis, pyelectasis, vesicoureteral reflux, and urinary tract infections, as well as an individual with unknown kidney abnormalities [21]. Soorya et al. (2013) analyzed 32 people with *SHANK3* deficiency, including terminal deletions of 22q13.3, 12 of whom had kidney disorders [5]. Types of renal abnormalities discussed were vesicoureteral reflux in 13%, hydronephrosis in 13%, renal agenesis in 6%, dysplastic kidney in 3%, and bilateral horseshoe kidneys and pyelectasis in 3%. The focus of these studies was on neurocognitive aspects of PMS rather than on kidney disorders. Samogy-Costa et al. (2019) [4] presented a large cohort of their own (n = 34) as well as analyses of separate cohorts from Tabet et al. (2017), [21] Lei et al. (2016), [47] and Soorya et al. (2013) [5]. Eight out of 34 subjects (23%) of Samogy-Costa et al.’s cohort had renal abnormalities that were not classified by type [4]. From the Disciglio et al. (2014) cohort, two of the nine individuals (22%) with interstitial deletions not involving the *SHANK3* gene had a kidney disorder [9]. One of the individuals had agenesis of the right kidney and enlargement of the left kidney, which was discovered through ultrasound and upon further evaluation was also determined to have vesicoureteral reflux. This child underwent surgery to correct the reflux at three years of age. The other individual’s kidney disorder was not stated. Palumbo et al. (2018) studied a 14-year-old male with a *de novo* interstitial deletion on 22q13.31 who was found to have renal hypoplasia [2]. When analyzing the DECIPHER database, the authors found 14 additional cases with similar deletions as the one detected in their case. Of these 14, five had known renal issues, which included renal dysplasia, renal agenesis, pyelectasis, and hydronephrosis [2]. Verhoeven et al. (2020) analyzed a cohort of 24 cases over the span of a decade [45]. Of those individuals, one presented with renal hypoplasia and the other with vesicoureteral reflux. Xu et al. (2020) looked at a cohort of 29 individuals with PMS from Mainland China: 21 of these were checked for kidney disorders, with three of them (14%) having documented kidney disorders [46]. Along with the cohort studies already discussed, some articles presented case studies.

### 3.2. Renal Disorders Assessed in Case Studies of Individuals with Phelan-McDermid Syndrome

Twelve case studies found in the literature reported kidney disorders in individuals with PMS (Table 2) [8,10,11,12,13,14,16,17,18,19,20,47]. Ismail et al. (2018) described a 16-year-old female who was referred after complaints of convulsions and renal problems. A pelvic ultrasound showed multiple cysts in the left kidney as well as bilateral hydronephrosis [14]. Kim et al. (2016) examined two infants with PMS and kidney disorders [16]. Multicystic kidney disease was noted in the 4-month-old female, and fetal pyelectasis in the 4-month-old male, both diagnosed through ultrasound. The female with multicystic kidney disease had normal renal function, thus no treatment was warranted. After a ventriculoperitoneal shunt was implanted, and prior to any renal treatment, the male with pyelectasis died at five months of age. Deibert et al. (2018) presented a 33-month-old female diagnosed with a multicystic kidney prenatally leading to a C-section delivery [8]. After delivery, a full renal workup was performed, and the child was found to have pyelectasis and hydronephrosis. Ha et al. (2017) presented two case studies with novel 22q13 deletions associated with clinical features distinct from PMS [12]. Case two also presented with renal cysts. Kirkpatrick et al. (2011) presented a case study of a three-year-old female with a 22q13 deletion, diagnosed via ultrasound at 22 weeks gestation with multicystic kidneys [17]. Periodic renal ultrasounds were performed to check for any changes as a follow-up. Lei et al. (2016) presented a case study of a six-year-old girl and her younger brother, both affected with kidney effusion and sharing a 22q13.31-q13.33 deletion [47]. Schröder et al. (1998) mapped deletions in three individuals, two of whom had kidney disorders [19]. These kidney disorders included bilateral enlarged kidneys in one and a polycystic right kidney detected by prenatal ultrasound in the other. The enlarged polycystic kidney led to complications in the perinatal period. Goizet et al. (2000) described a 14-year-old girl with vesicoureteral reflux and a duplicate kidney diagnosed through an ultrasound performed at five years of age due to a urinary tract infection and found to have a deletion on 22q13.3 [11]. Rowland et al. (2018) found renal cysts in a 54-year-old individual with a deletion on chromosome 22 after performing an MRI due to recurring urinary tract infections and stage one chronic kidney disease [18]. The individual was on lithium, which was tapered off after diagnosis of kidney disease. Fontes et al. (2015) presented a case with a deletion on 22q13.33 in which ultrasound revealed left kidney agenesis [10]. Ishikawa et al. (2015) presented a 5-year-old boy diagnosed with a multicystic dysplastic kidney and seizures [13]. Toruner et al. (2009) presented case studies of sudden infant death syndrome (SIDS), including one individual with a 22q13 deletion [20]. This infant was brought in for testing at the age of 10 weeks due to a prenatal ultrasound showing bilateral hydronephrosis and was found to have a 22q13 deletion. An intravenous pyelogram was then performed, which confirmed decreased functioning of the left kidney. The infant later succumbed to SIDS at 11 weeks of age. Terrone et al. (2017) [48] cited two individuals with PMS and renal abnormalities from other references [9,49] but showed no renal abnormalities in the case studies they presented.

A summary of the types of renal disorders in individuals with PMS as reported in the literature can be seen in Table 3. This combined the results from the cohorts and the case studies to provide a percentage of all renal disorders in PMS reported in the literature. The totals do not represent the total number of cases, as some individuals had more than one renal disorder.

### 3.3. Pooled Association Analysis

#### Sample Characteristics

**Figure 1:**
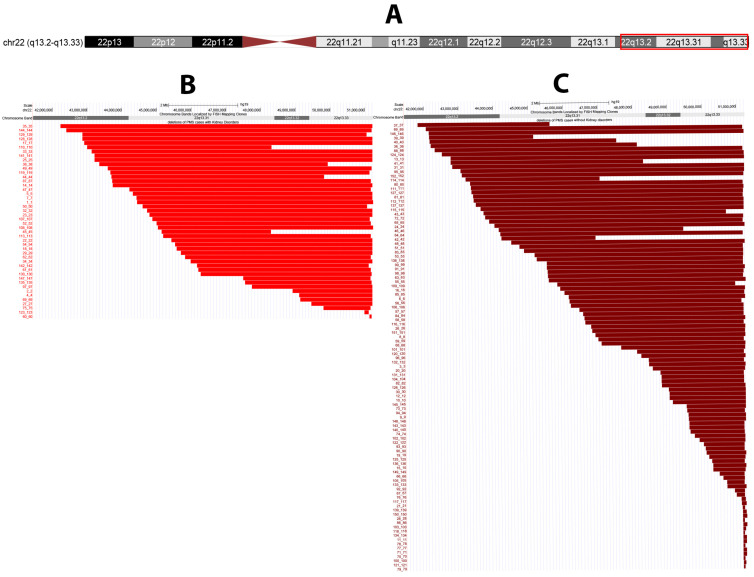
Deletions of people with Phelan-McDermid syndrome. Panel A depicts the chromosome 22q13 region of interest. Panel B presented deletions for individuals with PMS with reported kidney disorders and Panel C depicts deletions for individuals with PMS without reported kidney disorders on the right. Red bars show deleted regions. Figure created from UCSC Genome Browser [37,50]. There are more cases with PMS-*SHANK3*-unrelated in the group with kidney disorders.

A total of 152 subjects with PMS who had been evaluated for kidney disorders were collected from 13 publications [2,4,5,7,9,10,12,13,17,20,21,46,47] and were included in the pooled association analysis. (Supplemental Table S2) These subjects all had genomic deletion size or breakpoints provided in the manuscripts to allow association analysis (46 with renal disorders and 106 without renal disorders). The full dataset of individuals with chromosomal breakpoints can be seen in Supplemental File S1. Genotyping in the literature was achieved through multiple methods including: Illumina CytoSNP-12, Agilent (44K, 60K, 180K), Roche NimbleGen 135K, Illumina OmniExpress, and Affymetrix. The kidney disorder types, when reported, included renal cysts, renal hypoplasia or agenesis, hydronephrosis, vesicoureteral reflux, kidney dysplasia, horseshoe kidneys, and pyelectasis. The mean age of the pooled cohort was 8.81 years (range of 2.5 months to 45.4 years), and there were 74 males (49%) and 77 females (51%), with one unreported sex (Table 4). Ages and sex distribution were similar for those with and without kidney disorders (*p* > 0.05, Table 4). Individuals with kidney disorders had significantly larger deletions on average (mean 5.61 Mb, SD 2.28 Mb, range 8.92 Mb) than those without kidney disorders (mean 3.65, SD 2.84 Mb, range 9.06 Mb) (*p* = 1.78 × 10^−5^) (Figure 1, Table 4). The distribution of deletions is strikingly different, with most individuals with kidney disorders having deletions extending into chromosome bands 22q13.2-22q13.32. Patient 60 from Fontes et al. (2016) with a small deletion size of (71 KB) also had a large duplication of 16p13.3 that the authors considered the most likely cause of clinical features [10].

**Figure 2:**
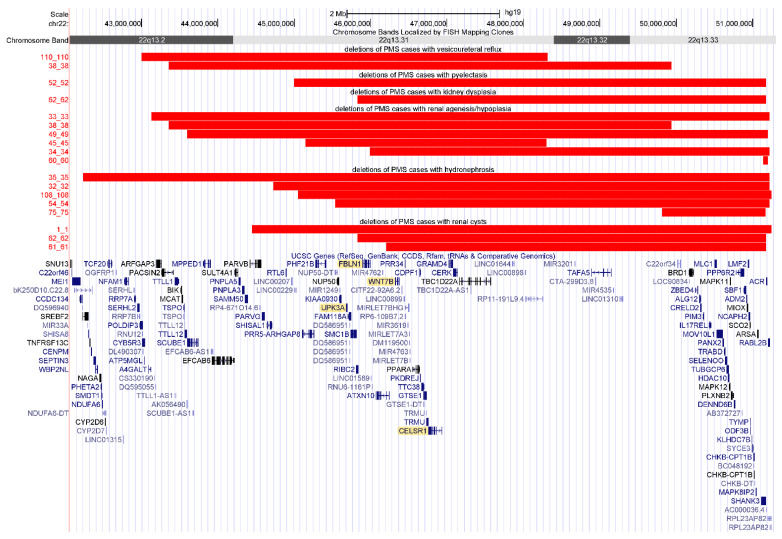
Deletions for individuals with PMS with renal disorders by type of disorder. Figure created from UCSC Genome Browser [37,50]. Red bars show the deleted regions. Renal disorders of unspecified type are not shown. Genes highlighted in yellow are candidate genes for renal disorders in PMS.

The distribution of deletions for those with kidney disorders was separated into each disorder subset (Figure 2). Segmental association analysis was conducted for the subset of individuals with identified renal disorders, but no regions were found to be statistically significant.

### 3.4. Association Analysis of Pooled Data

**Figure 3:**
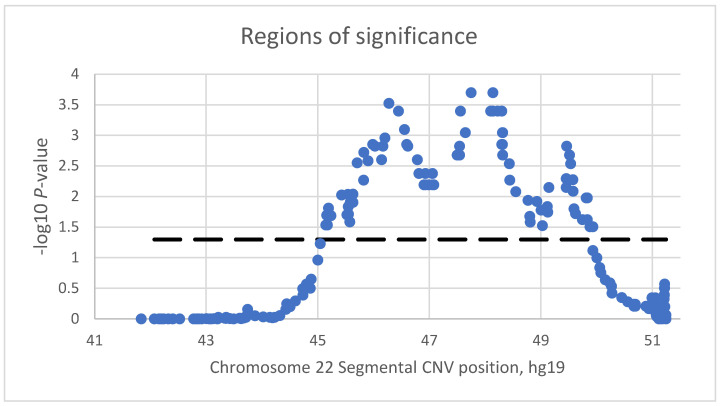
Association between genomic CNV regions on 22q13 and kidney disorders. The dashed line corresponds to a *p* = 0.05.

The prevalence of kidney disorders and the segmental CNV-identified regions were analyzed by plotting the −log_10_ *p*-value against the deletion position along chromosome 22q13 (Figure 3). The CNV segmental association analysis identified a genomic region from 45.16 Mb to 49.93 Mb that was significantly associated with having a kidney disorder (Figure 3). A total of 38 genes are located in these regions. The regions from position 45.16 to 47.05 Mb, position 47.54 to 49.00 Mb, and position 49.46 to 49.81 Mb were significantly associated with kidney disorders and showed peaks of association near positions 47.75 Mb, 48.14 Mb, and 49.46 Mb. Genes within the CNV segments associated with kidney disorders at *p* < 0.05 as well as chromosome locations from GeneCards [51] are provided in Supplemental Table S3. The genes *UPK3A*, *FBLN1*, *WNT7B*, and *CELSR1* have the strongest evidence of association with kidney disorders.

## 4. Discussion

We found that 9–40% (mean of 22%) of individuals with PMS in the literature with 22q13 deletions had kidney disorders. Types of reported renal disorders included agenesis/hypoplasia, kidney cysts, hydronephrosis, vesicoureteral reflux, kidney dysplasia, and pyelectasis. Individuals with kidney disorders tended to have large deletions (mean 5.49 Mb) or to encompass the region from 45.16 Mb to 49.93 Mb as an interstitial deletion or a terminal deletion of 1.31 Mb–6.08 Mb. Using association analysis, candidate gene prioritization based upon a set of known kidney-related genes and estimates of haploinsufficiency, we identified *UPK3A*, *FBLN1*, *WNT7B*, and *CELSR1* as priority candidate genes for kidney disorders.

Several investigators have sought to identify candidate genes for renal disorders in PMS. Samogy-Costa et al. (2019) gave support to *UPK3A*, *ZBED4*, *CELSR1*, and *FBLN1* being candidate genes [4]. Ricciardello et al. (2021) suggested *CELSR1* and *UPK3A* as candidates for urogenital anomalies [52]. Palumbo et al. (2018) noted that *TRMU* and *PPARA* are expressed in the kidney [2]. Others have proposed *FBLN1*, *WNT7B*, *CELSR1*, and *ZBED4* as potential candidate genes that influence kidney development [53,54]. *ZBED4* was outside the region found to be significant in this data set. *SHANK3* is the primary gene associated with neurobehavioral features of PMS but was not in the region associated with kidney disorders in this investigation. It is unlikely to be associated with kidney disorders based on the lack of renal disorders observed in individuals with the smallest 22q13.3 terminal deletions or carrying *SHANK3* pathogenic variants [4,6].

The proposed candidate genes have varying degrees of supporting evidence in addition to being in the genomic region found most associated with kidney disorders. Mouse models with deletion of *FBLN1* show abnormal kidney development [55]. WNT signaling, including from WNT7B, is involved in kidney development [56]. *UPK3A* may be involved in vesicoureteral reflux [57]. *CELSR1* is involved in kidney patterning [58,59]. The review by Ricciardello et al. (2021) highlighted the genes *UPK3A*, *CELSR1*, *SCUBE1*, *PLXNB2*, and *FBLN1* as being potentially associated with kidney disorders in the region upon which we focused [52]. Outside our focus region, they also suggest *RABL2B*, *PNPLA3*, and *SCUBE* as associated genes. A recent classification of PMS has been proposed which distinguishes between *SHANK3*-unrelated and *SHANK3*-related cases, depending on the involvement of *SHANK3* in deletions or pathogenic variants [60]. In our study, we noted that kidney disorders were not reported as frequently in cases with small 22q13 terminal deletions. These findings support the hypothesis that *SHANK3* does not play a major role in kidney disorders in PMS. Agreeing with these findings is the recent study by Nevado et al. (2022) on a cohort of n = 210 who reported that 22% of people with PMS had a renal disorder, that renal disorders were associated with larger deletion sizes, and only 1 case of renal disorder was observed in the group with *SHANK3* variants [61].

## 5. Limitations

This investigation included a review of the literature on kidney disorders in PMS and statistical analysis of a large sample size created by pooling data from peer-reviewed literature to identify genomic regions associated with kidney disorders. There were limitations in this investigation. Many cases did not specify the type of kidney disorder present, reducing the specificity of the analyses. The pathogenic mechanism leading to different kidney disorders may involve distinct genes on 22q13, and by combining all kidney disorders, the identification of specific causal genes may have been blurred. For instance, renal anomalies originating from abnormal development (renal agenesis, hypoplasia, horseshoe kidney, pyelectasis, kidney dysplasia) may have distinct genomic origins compared to renal disorders developing later in life (renal cysts, hydronephrosis, vesicoureteral reflux). The fewer numbers of cases with specific diagnoses made statistical analysis underpowered for the assessment of specific diagnoses. Not all manuscripts reporting PMS cases assessed patients for kidney disorders, and thus, the prevalence of kidney disorders may be under- or over-estimated. The genetic data came from multiple testing platforms of varying levels of sensitivity, and in some cases, deletions were estimated based upon reported deletion sizes.

## 6. Conclusions

Kidney disorders are relatively common in Phelan-McDermid syndrome, occurring in approximately 9–40% of individuals [2,3,4,5,7,9,15,21,45,46]. Individuals with PMS should be evaluated for kidney disorders for improved clinical management [1,62], particularly those with interstitial deletions or terminal deletions greater than >4 Mb in size. The results from this study suggest that *UPK3A*, *FBLN1*, *WNT7B*, and *CELSR1* are strong candidate genes for kidney disorders in PMS and merit functional studies. Future clinical assessments of people with PMS should include assessment of the renal system.

## Figures and Tables

**Table 1:** Summary of kidney disorders found in Phelan-McDermid syndrome cohort studies.

Cohort Study	Type of Kidney/Renal Disorders Reported in at Least Individuals	Genes Proposed to Be Associated with Kidney/Renal Disorders	Number of Cases	Number with Renal Disorder	% with Renal Disorder
Bonaglia et al., 2011 [7]	Hydronephrosis, renal agenesis, hypoplasia		44	4	9%
Disciglio et al., 2014 [9]	Renal agenesis, vesicoureteral reflux, other renal issues		9	2	22%
Jefferies et al., 2005 [15]	Vesicoureteral reflux, multicystic kidney, other renal issues	*FBLN1*, *WNT7B*	30	5	17%
Palumbo et al., 2018 [2]	Renal hypoplasia, renal dysplasia, renal agenesis, pyelectasis, hydronephrosis, other renal issues	*PHF21B*, *GRAMD4*, *TRMU*, *FBLN1*, *WNT7B*, *PPARA*	15	6	40%
Sarasua et al., 2014 [3]	Hydronephrosis, pyelectasis, polycystic kidneys, vesicoureteral reflux		148	39	26%
Samogy-Costa et al., 2019 [4]	Not reported	*ZBED4*, *CELSR1*, *FBLN1*, *UPK3A*	34	8	23%
Soorya et al., 2013 [5]	Vesicoureteral reflux, hydronephrosis, renal agenesis, dysplastic kidney, horseshoe kidney, pyelectasis		32	12	37%
Tabet et al., 2017 [21]	Multicystic kidney, renal dysplasia, hydronephrosis, pyelectasis, vesicoureteral reflux, other renal issues		78	7	9%
Verhoeven et al., 2019 [45]	Renal hypoplasia, vesicoureteral reflux		24	2	8%
Xu et al., 2020 [46]	Not reported		29	3	10%

**Table 2:** Summary of kidney disorders found in Phelan-McDermid syndrome case studies.

Case Study	Type of Renal Disorders	Individuals Assessed for Renal Disorders	Number with Renal Disorders
Deibert et al., 2019 [8]	Multicystic kidney, pyelectasis, hydronephrosis	1	1
Fontes et al., 2015 [10]	Agenesis	1	1
Goizet et al., 2000 [11]	Vesicoureteral reflux, other renal issues	1	1
Ha et al., 2016 [12]	Multicystic kidney	2	1
Ishikawa et al., 2015 [13]	Multicystic kidney	1	1
Ismail et al., 2018 [14]	Multicystic kidney, hydronephrosis	2	1
Kim et al., 2016 [16]	Multicystic kidney, pyelectasis	2	2
Kirkpatrick et al., 2011 [17]	Multicystic kidney	1	1
Lei et al., 2016 [47]	Other renal issues	1	1
Rowland et al., 2018 [18]	Multicystic kidney	2	1
Schröder et al., 1998 [19]	Multicystic kidney, other renal issues	3	2
Toruner et al., 2009 [20]	Hydronephrosis	1	1

**Table 3:** Summary of types of renal disorders in individuals with Phelan-McDermid Syndrome as reported in the literature.

Disorder	Renal Disorders Reported in Case Reports	Renal Disorders Reported in Cohorts	% of All Renal Disorders in PMS Reported in Literature
Renal cysts	9	8	16%
Renal agenesis/hypoplasia	1	7	7%
Hydronephrosis	3	19	20%
Vesicoureteral reflux	1	27	26%
Kidney dysplasia	0	4	4%
Horseshoe kidney	0	1	1%
Pyelectasis	2	10	11%
Other/Unknown	1	18	17%
Total renal disorders reported *	17	94	100%

* The totals do not represent the total number of cases, as some individuals had more than one renal disorder.

**Table 4:** Characteristics of the study population (n = 152) and association with deletion size means.

	With Kidney Disorder	Without Kidney Disorder	*p*-Value
Total count	46 (30.3%)	106 (69.7%)	N/A
Females/Males	25/20	52/54	0.5339
Mean age, years	7.00	8.68	0.3527
Mean (SD) size of all deletions, Mb	5.61 (2.28)	3.65 (2.84)	1.78 × 10^−5^
Mean (SD) size of deletions for those with agenesis/hypoplasia deletions, Mb (N = 6)	4.35 (3.44)	N/A	
Mean (SD) size of deletions for those with renal cysts, Mb (N = 3)	5.71 (0.96)	N/A	
Mean (SD) size of deletions for those with vesicoureteral reflux, Mb (N = 2)	5.94 (0.89)	N/A	
Mean (SD) size of deletions for those with pyelectasis, Mb (N = 1)	6.17 (N/A)	N/A	
Mean (SD) size of deletions for those with kidney dysplasia, Mb (N = 1)	6.95 (1.13)	N/A	
Mean (SD) size of deletions for those with hydronephrosis, Mb (N = 5)	7.74 (3.17)	N/A	
Mean (SD) size of deletions for those with unspecified kidney disorders, Mb (N = 28)	6.04 (2.20)	N/A	

## Data Availability

The data presented in this study are available in the Supplementary Materials.

## Supplementary Materials

The following supporting information can be downloaded at: https://www.mdpi.com/article/10.3390/genes13061042/s1, Table S1. Genes known to be associated with renal abnormalities used as a training set for ToppGene; Table S2. Source of patient data for the pooled analysis (N = 152); Table S3. Genes within the CNV segments associated with kidney disorders at p < 0.05 with gene location, prioritization rank out of 38 genes, HI < 10% estimate, and pLi score if >0.9 from gnomAD; Supplemental File S1. Dataset of 152 people with PMS evaluated for kidney disorders abstracted from the published literature.
