# Supplementary material for: State of the Science for Kidney Disorders in Phelan-McDermid Syndrome: UPK3A, FBLN1, WNT7B, and CELSR1 as Candidate Genes
Source: Genes (Basel). 2022 Jun 10;13(6):1042. doi: 10.3390/genes13061042 (PMC9223119; doi:10.3390/genes13061042)
Supplement: Supplementary file 1 [file genes-13-01042-s001.zip › genes-1747977-supplementary/new supple/McCoy.State of the Science Kidney PMS_Genes_Supplemental Tables May 12 2022.pdf]

Supplemental Tables

McCoy et al. State of the Science for Kidney Disorders in Phelan-McDermid Syndrome 2022

Supplemental Table S1. Genes known to be associated with renal abnormalities used as a training set with ToppGene [1-5]

Gene	Kidney dysplasia	Renal hypoplasia	Renal cysts or Cystic disease	Renal agenesis	Vesicoureteral reflux	Renal hypodysplasia
BMP4						✓
CHDIL					✓	✓
DICER1			✓			
DSTYK						✓
EYA1		✓				
FRAS1				✓		
FREM2				✓		
GATA3	✓					
GDNF		✓		✓		
GREB1L		✓		✓		
HNF1B	✓	✓	✓			
ITGA8		✓		✓		
KAL1/ANOS1				✓		
MUC1			✓			
PAX2	✓	✓				
PKD1, PKD2			✓			
RET		✓		✓		
ROBO2					✓	
SALL1						✓
SEMA3A	✓					
SIX1, SIX2, SIX5					✓	✓
SOX17					✓	
TNXB					✓	
TRAP1				✓	✓	
UMOD			✓			
UPK3A		✓		✓		
VUR1					✓	
WNT4						✓

Supplemental Table S2. Source of patient data for the pooled analysis (N=152)

Study	***Patients included in the study***
**Kirkpatrick et al, 2011** [6]	1
**Soorya et al, 2013** [7]	30
**Bonaglia et al, 2011** [8]	4
**Disciglio et al, 2014** [9]	9
**Palumbo et al, 2017** [10]	15
**Fontes et al, 2015** [11]	1
**Ishikawa et al, 2015** [12]	1
**Tabet et al, 2017** [13]	46
**Lei et al, 2016** [14]	1
**Toruner et al, 2009** [15]	1
**Ha et al, 2017** [16]	1
**Samogy-Costa et al, 2019** [17]	27
**Xu et al, 2020** [18]	15

Supplemental Table S3. Genes within the CNV segments associated with kidney disorders at *P*<0.05 with gene location, prioritization rank out of 38 genes, HI<10% estimate [19], and pLI score if >0.9 from gnomAD [20].

Gene	***Empirical P-value***	***Gene location***	**Prioritization rank**	***pLI >0.9 or HI <10%***
PRR5	0.034997	chr22:45,064,427-45,133,561	15	
PRR5-ARHGAP8	0.020698	chr22:45,098,113-45,258,664	5	
ARHGAP8	0.020698	chr22:45,098,355-45,258,665	12	
PHF21B	0.013199	chr22:45,277,042-45,405,880	21	pLI 0.99
NUP50	0.018398	chr22:45,559,722-45,583,896	13	
KIAA0930	0.007899	chr22:45,586,219-45,636,650	23	
MIR1249	0.007899	chr22:45,596,835-45,596,900	26	
UPK3A*	**0.005399**	**chr22:45,680,830-45,691,755**	**4**	
FAM118A	0.0028	chr22:45,704,849-45,737,836	24	
SMC1B	0.0028	chr22:45,739,944-45,809,500	11	
RIBC2	0.0017	chr22:45,809,572-45,828,376	27	
FBLN1	**0.0009**	**chr22:45,898,118-45,997,015**	**2**	
ATXN10	0.0005	chr22:46,067,678-46,241,187	8	
MIR4762	0.002	chr22:46,156,404-46,156,478	33	
WNT7B	**0.0003**	**chr22:46,316,242-46,373,009**	**1**	
LOC730668	0.0003	chr22:46,402,496-46,406,657	37	
LINC00899	0.0003	chr22:46,435,787-46,440,748	36	
PRR34	0.0003	chr22:46,445,358-46,450,024	20	
MIRLET7BHG	0.0003	chr22:46,449,749-46,509,808	22	
MIR3619	0.0003	chr22:46,486,924-46,487,006	30	

MIRLET7A3	0.0003	chr22:46,508,629-46,508,702	18	
MIR4763	0.0003	chr22:46,509,446-46,509,537	38	
MIRLET7B	0.0003	chr22:46,509,566-46,509,648	7	
PPARA	0.0012	chr22:46,546,424-46,639,653	3	HI 7.3%
CDPF1	0.0012	chr22:46,639,908-46,646,576	25	
PKDREJ	0.0012	chr22:46,651,560-46,659,219	9	
TTC38	0.0012	chr22:46,663,858-46,689,905	28	
GTSE1	0.0012	chr22:46,692,638-46,726,707	10	
TRMU	0.0012	chr22:46,726,772-46,753,237	16	
CELSR1	**0.004**	**chr22:46,756,731-46,933,067**	**6**	**pLI 1.00**
GRAMD4	0.004	chr22:46,971,909-47,075,688	19	pLI 1.00
CERK	0.004	chr22:47,080,307-47,134,158	17	
TBC1D22A	0.0005	chr22:47,158,518-47,571,342	14	
LINC00898	0.0002	chr22:48,016,792-48,027,318	36	
MIR3201	0.005199	chr22:48,670,176-48,670,227	31	
LOC284933	0.006699	chr22:48,934,712-48,943,199	32	
MIR4535	0.0042	chr22:49,176,107-49,176,165	35	
LINC01310	0.0042	chr22:49,262,582-49,294,198	34	

**UPK3A* is already a known kidney-related gene.

References

[1] Bertoli Avella, A.; Conte, M.L.; Punzo, F.; Graaf, B.; Lama, G.; La Manna, A.; Polito, C.; Grassia, C.; Nobili, B.; Rambaldi, P.F. *et al.* ROBO2 Gene Variants are Associated with Familial Vesicoureteral Reflux. American Society of Nephrology.Journal **2008**, *19*, 825-831.

[2] Hu, F.; Sun, L. Recognizable Type of Pituitary, Heart, Kidney and Skeletal Dysplasia mostly Caused by SEMA3A Mutation: A Case Report. World J Clin Cases **2019**, *7*, 3310-3315.

[3] Humbert, C.; Silbermann, F.; Morar, B.; Parisot, M.; Zarhrate, M.; Masson, C.; Tores, F.; Blanchet, P.; Perez, M.; Petrov, Y. *et al.* Integrin Alpha 8 Recessive Mutations are Responsible for Bilateral Renal Agenesis in Humans. Am J Hum Genet **2014**, *94*, 288-294.

[4] Brophy, P.D.; Rasmussen, M.; Parida, M.; Bonde, G.; Darbro, B.W.; Hong, X.; Clarke, J.C.; Peterson, K.A.; Denegre, J.; Schneider, M. *et al.* A Gene Implicated in Activation of Retinoic Acid Receptor Targets is a Novel Renal Agenesis Gene in Humans. Genetics **2017**, *207*, 215-228.

[5] Vivante, A.; Kohl, S.; Hwang, D.; Dworschak, G.C.; Hildebrandt, F. Single-Gene Causes of Congenital Anomalies of the Kidney and Urinary Tract (CAKUT) in Humans. Pediatr. Nephrol. **2014**, *29*, 695.

[6] Kirkpatrick, B.E.; El-Khechen, D. A Unique Presentation of 22q13 Deletion Syndrome: Multicystic Kidney, Orofacial Clefting, and Wilms' Tumor. Clin Dysmorphol **2011**, *20*, 53-54.

[7] Soorya, L.; Kolevzon, A.; Zweifach, J.; Lim, T.; Dobry, Y.; Schwartz, L.; Frank, Y.; Wang, A.T.; Cai, G.; Parkhomenko, E. *et al.* Prospective Investigation of Autism and Genotype-Phenotype Correlations in 22q13 Deletion Syndrome and SHANK3 Deficiency. Molecular autism **2013**, *4*, 18.

[8] Bonaglia, M.C.; Giorda, R.; Beri, S.; De Agostini, C.; Novara, F.; Fichera, M.; Grillo, L.; Galesi, O.; Vetro, A.; Ciccone, R. *et al.* Molecular Mechanisms Generating and Stabilizing Terminal 22q13 Deletions in 44 Subjects with Phelan/McDermid Syndrome. PLoS Genet **2011**, *7*, e1002173.

[9] Disciglio, V.; Lo Rizzo, C.; Mencarelli, M.A.; Mucciolo, M.; Marozza, A.; Di Marco, C.; Massarelli, A.; Canocchi, V.; Baldassarri, M.; Ndoni, E. *et al.* Interstitial 22q13 Deletions Not Involving SHANK3 Gene: A New Contiguous Gene Syndrome. Am J Med Genet A **2014**, *164A*, 1666-1676.

[10] Palumbo, P.; Accadia, M.; Leone, M.P.; Palladino, T.; Stallone, R.; Carella, M.; Palumbo, O. Clinical and Molecular Characterization of an Emerging Chromosome 22q13.31 Microdeletion Syndrome. American Journal of Medical Genetics Part A **2018**, *176*, 391-398.

[11] Fontes, M.I.B.; Santos, A.P.; Molck, M.C.; Simioni, M.; Nascimento, D.L.L.; Andrade, A.K.M.; Rosenberg, C.; Krepischi, A.C.V.; Appenzeller, S.; Monlleó, I.L. *et al.* Genotype-phenotype Correlation of 16p13.3 Terminal Duplication and 22q13.33 Deletion: Natural History of a Patient and Review of the Literature. American Journal of Medical Genetics Part A **2016**, *170*, 766-772.

[12] Ishikawa, N.; Kobayashi, Y.; Fujii, Y.; Yamamoto, T.; Kobayashi, M. Late-Onset Epileptic Spasms in a Patient with 22q13.3 Deletion Syndrome. Brain Dev. **2015**, *38*, 109-112.

[13] Tabet, A.; Rolland, T.; Ducloy, M.; Lévy, J.; Buratti, J.; Mathieu, A.; Haye, D.; Perrin, L.; Dupont, C.; Passemard, S. *et al.* A Framework to Identify Contributing Genes in Patients with Phelan-McDermid Syndrome. NPJ Genom Med **2017**, *2*, 32.

[14] Lei, D.; Li, S.; Banerjee, S.; Zhang, H.; Li, C.; Hou, S.; Chen, D.; Yan, H.; Li, H.; Peng, H.H. *et al.* Clinical and Genomic Evaluation of a Chinese Patient with a Novel Deletion Associated with Phelan-McDermid Syndrome. Oncotarget **2016***, 7*, 80327-80335.

[15] Toruner, G.A.; Kurvathi, R.; Sugalski, R.; Shulman, L.; Twersky, S.; Pearson, P.G.; Tozzi, R.; Schwalb, M.N.; Wiley, Wallerstein R. Copy number variations in three children with sudden infant death. Clin Genet 2009: 76: 63–68. John; Sons, A. *et al.* Copy Number Variations in Three Children with Sudden Infant Death. **2009**.

[16] Ha, J.F.; Ahmad, A.; Lesperance, M.M. Clinical Characterization of Novel Chromosome 22q13 Microdeletions. Int. J. Pediatr. Otorhinolaryngol. **2016***, 95*, 121-126.

[17] Samogy-Costa, C.I.; Varella-Branco, E.; Monfardini, F.; Ferraz, H.; Fock, R.A.; Barbosa, R.H.A.; Pessoa, A.L.S.; Perez, A.B.A.; Lourenço, N.; Vibranovski, M. *et al.* A Brazilian Cohort of Individuals with Phelan-McDermid Syndrome: Genotype-Phenotype Correlation and Identification of an Atypical Case. J Neurodev Disord **2019***, 11*, 13.

[18] Xu, N.; Lv, H.; Yang, T.; Du, X.; Sun, Y.; Xiao, B.; Fan, Y.; Luo, X.; Zhan, Y.; Wang, L. *et al.* A 29 Mainland Chinese Cohort of Patients with Phelan–McDermid Syndrome: Genotype–phenotype Correlations and the Role of SHANK3 Haploinsufficiency in the Important Phenotypes. Orphanet J Rare Dis **2020***, 15*.

[19] Huang, N.; Lee, I.; Marcotte, E.M.; Hurles, M.E. Characterising and Predicting Haploinsufficiency in the Human Genome. PLoS Genet **2010***, 6*, e1001154.

[20] Karczewski, K.J.; Francioli, L.C.; Tiao, G.; Cummings, B.B.; Alföldi, J.; Wang, Q.; Collins, R.L.; Laricchia, K.M.; Ganna, A.; Birnbaum, D.P. *et al.* The Mutational Constraint Spectrum Quantified from Variation in 141,456 Humans. Nature **2020***, 581*, 434-443.
